# Characterization of WAC interactions with R2TP and TTT chaperone complexes linking glucose and glutamine availability to mTORC1 activity

**DOI:** 10.1002/2211-5463.70085

**Published:** 2025-07-13

**Authors:** Sofía Cabezudo, Natalia Cuervo, Carmen García‐Martín, Andrés López‐Perrote, Clara Reglero, Adrián Maqueda‐Real, Ana González‐Corpas, Marina Serna, Diego Megías, Alejo Efeyan, Solip Park, Oscar Llorca

**Affiliations:** ^1^ Structural Biology Programme Spanish National Cancer Research Centre (CNIO) Madrid Spain; ^2^ Confocal Microscopy Unit Spanish National Cancer Research Centre (CNIO) Madrid Spain; ^3^ Molecular Oncology Programme Spanish National Cancer Research Centre (CNIO) Madrid Spain; ^4^ Present address: Advanced Optical Microscopy Unit, UCCTs Instituto de Salud Carlos III (ISCIII) Madrid Spain

**Keywords:** mTORC1, R2TP, RUVBL1‐RUVBL2, TELO2, WAC

## Abstract

TELO2‐TTI1‐TTI2 (TTT) and R2TP are multi‐subunit chaperones that cooperate with HSP90 to assemble matured complexes of the PIKK family of kinases, including mTOR complex 1 (mTORC1). WAC, a protein previously implicated in transcription, H2B ubiquitination, and autophagy, was recently identified as a regulator of mTORC1 in response to glucose and glutamine availability, acting in concert with R2TP and TTT. However, the molecular basis of the interactions of WAC with R2TP and TTT and their role in mTORC1 regulation remains poorly defined. Here, we characterized the interactions of WAC with mTOR, R2TP, and TTT and how these are affected by nutrient conditions. Using purified proteins, we establish that WAC directly binds to mTOR‐mLST8, R2TP, and TELO2, but not TTI1 and TTI2. In cells, WAC is part of complexes containing components of mTORC1, R2TP, and TTT, and these associations are modulated by nutrient availability. Notably, WAC and TELO2 strongly associate with mTOR under glucose and glutamine deprivation, and these interactions are weakened minutes after nutrient refeeding. These dynamics correlate with changes in mTORC1 activity. Transcriptomic and proteomic analysis shows that WAC, mTOR, R2TP, and TTT are co‐expressed across several human cancers, supporting that WAC is part of a functional pathway with mTOR, R2TP, and TTT. Together, our findings reveal the formation and disassembly of a WAC complex with mTOR and TELO2 that contributes to regulate mTORC1 in response to glucose and glutamine availability.

## Graphical abstract

TTT and R2TP chaperone complexes are required for the assembly and activation of mTORC1. WAC directly interacts with components of TTT, R2TP, and mTORC1, and these interactions are affected by the availability of glucose and glutamine, correlating with changes in mTORC1 activity. Thus, the interaction of WAC with TTT and R2TP could contribute to regulating mTORC1 in response to nutrients.

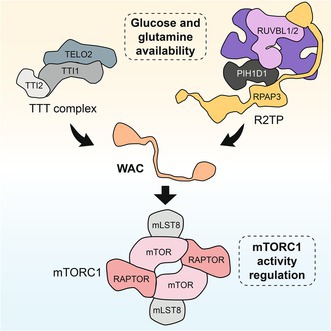

**Abbreviations**
CPTAC: Clinical Proteomic Tumor Analysis Consortium
CRISPR/Cas9: clustered regularly interspaced short palindromic repeats/CRISPR‐associated protein 9
GST: glutathione S‐transferase
HEK293T: human embryonic kidney 293T cells
HSP90: heat shock protein 90
mLST8: mammalian lethal with SEC13 protein 8
mTOR: mechanistic target of rapamycin
mTORC1: mTOR complex 1
PAQosome: particle for arrangement of quaternary structure
PIH1D1: protein interacting with HSP90 1 domain‐containing protein 1
R2TP: RUVBL1‐RUVBL2‐Tah1‐Pih1 chaperone complex
RAPTOR: regulatory‐associated protein of mTOR
RPAP3: RNA polymerase II‐associated protein 3
RUVBL1: RuvB‐like 1
RUVBL2: RuvB‐like 2
TELO2: telomere maintenance 2 protein
TTI1: TELO2‐interacting protein 1
TTI2: TELO2‐interacting protein 2
TTT: TELO2‐TTI1‐TTI2 complex
WAC: WW domain‐containing adaptor with coiled‐coil

The Rvb1‐Rvb2‐Tah1‐Pih1 (R2TP) complex is a heat shock protein 90 (HSP90) cochaperone made up of four subunits that received its name from the composition of subunits in yeast. In humans, R2TP is organized by the interaction of two subcomplexes, RUVBL1‐RUVBL2 and RPAP3‐PIH1D1. RUVBL1 and RUVBL2, the metazoan homologs of yeast Rvb1 and Rvb2, are two related AAA‐ATPases forming a heterohexameric ring, whereas RPAP3 (Tah1 in yeast) contains two tetratricopeptide repeat (TPR) domains that interact with HSP90 [1, 2]. PIH1D1 contains a PIH domain that preferentially binds to the phosphorylated version of a short consensus sequence found in several proteins including TELO2, a protein that collaborates with R2TP in the maturation of a specific set of clients (see next paragraph) [3, 4]. R2TP is involved in the biogenesis and assembly of large complexes such as spliceosomal U4 and U5 snRNPs, snoRNP complexes of the L7Ae family such as telomerase, and RNA polymerase II [5, 6, 7].

R2TP works in concert with the TTT complex, which is composed of TELO2, TTI1, and TTI2 [8], during the maturation of the complexes formed by the phosphatidylinositol 3‐kinase (PI3K)‐like kinases (PIKKs), a family that includes ataxia telangiectasia‐mutated and Rad3‐related (ATR), mechanistic target of rapamycin (mTOR), ataxia telangiectasia mutated (ATM), and suppressor of morphogenesis in genitalia‐1 (SMG1), among others [9, 10]. TTT and R2TP are known to interact [11] and both complexes, together with HSP90, are essential for the assembly of the ATR‐interacting protein (ATRIP) complex, the mTOR complexes mTORC1 and mTORC2, and the complex formed by SMG1 with SMG8 and SMG9 [7].

Current models propose that TTT protects the nascent polypeptide chains of PIKK kinases, including mTOR, from misfolding and aggregation, thereby promoting their cotranslational maturation and proper folding [12]. In the case of mTOR, the TTT complex also interacts with R2TP to deliver the kinase. Then, R2TP acts in concert with HSP90 to promote the incorporation of regulatory‐associated protein of mTOR (RAPTOR) to mTOR. R2TP is thought to facilitate that HSP90 engages mTOR and promotes the incorporation of other subunits of mTORC1 and its dimerization [5, 10], but the mechanisms are still not elucidated [4, 8, 9, 11].

The core R2TP machinery needs additional factors to recruit specific clients and to regulate the chaperone complex in response to specific signals. For instance, maturation of the RNA polymerase II involves the interaction of R2TP with a complex containing the unconventional Prefoldin RPB5 interactor 1 (URI1), a protein that is part of the prefoldin‐like (PFDL) complex [13]. It has been proposed that R2TP and PFLD form a unique mega‐assembly, named the PAQosome (particle for arrangement of quaternary structure), and which could serve as a chaperone for the maturation of many multiprotein complexes [6, 14]. However, the function of the R2TP/PFLD complex is only well‐characterized in the case of the cytosolic assembly of RNA polymerase II, raising the possibility that this interaction is needed for the maturation of a specific subset of complexes [14]. In the case of mTORC1, WAC (WW domain‐containing Adaptor with Coiled‐Coil) has been proposed to promote the interaction between TTT and R2TP, and mTORC1 dimerization [15]. However, how accessory factors interact and regulate the R2TP machinery for specific clients has been poorly studied [6, 7].

mTORC1 integrates information from cellular energy, nutrients, growth factors, and various forms of stress to initiate a growth regime when conditions are favorable [16]. mTORC1 complex is formed by a dimeric structure containing two copies of mTOR, mammalian lethal with SEC13 protein 8 (mLST8) and RAPTOR [17, 18]. mLST8 stabilizes the kinase domain of mTOR [19], whereas RAPTOR is essential for mTORC1 dimerization, proper subcellular localization, and recruitment of canonical mTORC1 substrates [20, 21].

Several studies have described the mechanisms by which the recruitment and activation of mTOR occurs at a molecular and structural level in response to growth factors and other mitogens [22, 23, 24], and amino acids [22, 25, 26]. Emerging evidence points to additional means by which glucose and various amino acids regulate mTORC1 activity [27, 28, 29, 30, 31], indicating that multiple pathways and signals intervene to regulate the activity of mTORC1. In this scenario, a few reports have shown that mTORC1 can also be regulated by affecting the activity of the TTT‐R2TP chaperone system [15, 32, 33]. For instance, Kim *et al*. found that deprivation of glucose and glutamine reduces the interaction between RUVBL1‐RUVBL2 and TTT, disrupts mTORC1 homodimerization and proper lysosomal localization and activation [33]. The authors suggested that there is a mechanism that interferes with the formation of the RUVBL1‐RUVBL2‐TTT complex, and this affects mTORC1 assembly under deprivation of glutamine and glucose.

Notably, David‐Morrison *et al*. observed that endogenous WAC interacted with mTOR and components of the R2TP and TTT complexes [15]. They interpreted all their results as consistent with a model where WAC serves as an adaptor promoting the interaction between the TTT and R2TP systems and therefore facilitating the incorporation of RAPTOR to mTOR and mTORC1 dimerization in response to energy [15]. WAC had been previously implicated in the regulation of different biological processes such as mitotic entry [34], H2B ubiquitination [35], and autophagy [36, 37]. In each of these pathways, WAC forms complexes with different proteins.

Despite recent work, our understanding of how WAC recognizes the R2TP and TTT chaperone complexes to regulate mTORC1 activity remains limited and is, to our knowledge, largely based on the work by David‐Morrison *et al*. [15]. In this study, we characterized the interactions between WAC, mTOR, and the R2TP and TTT chaperone complexes to gain mechanistic and molecular insights into how WAC regulates mTORC1 activity. Our results suggest the formation and disassembly of a complex containing WAC, mTOR, and TELO2 in response to nutrient conditions that correlate with changes in mTORC1 activity.

## Materials and methods

### Cloning

Constructs for recombinant protein expression were generated by using the IVA cloning protocol [38]. The cDNA of human WAC (NM_016628.5) was obtained from GenScript USA Inc (Piscataway, NJ, USA) (clone ID OHu28471) and cloned into the pACEMam vector [kindly provided by Dr Rafael Fernández‐Leiro (CNIO, Spain)] including a C‐terminal 3xFlag tag with Human Rhinovirus (HRV) 3C protease site (pACEMam‐WAC‐3C‐3xFlag). The cDNAs of human TELO2 (NM_016111), TTI1 (NM_014657.3), and TTI2 (NM_001102401.3) were obtained from the CNIO Genomics Unit cDNA clone library (CNIO, Spain). TELO2 was cloned into the pACEBac1 vector [kindly provided by Dr Rafael Fernández‐Leiro (CNIO, Spain)] including an N‐terminal GST tag followed by Tobacco Etch Virus (TEV) protease site (pACEBac1‐GST‐TEV‐TELO2), while a dual TTI1‐TTI2 construct was inserted into pACEBac1, including an N‐terminal Twin‐Strep tag, followed by a 3xHA tag and an HRV 3C protease site in TTI1 (pACEBac1‐TwinStrep‐3xHA‐3C‐TT1‐TT2). A ready‐to‐express construct containing an N‐terminal 10xHis‐tagged mTOR with TEV protease site and untagged mLST8 was purchased from GenScript for co‐expression of both proteins in insect cells (pACEBac1‐10xHis‐TEV‐mTOR‐mLST8). Ready‐to‐express constructs of RPAP3 (NM_001146075.2) cloned into a pRSFDuet‐1 vector containing an N‐terminal 10xHis‐SUMO‐tag with TEV protease site and a C‐terminal Strep‐II‐tag with HRV 3C site (pRSFDuet1‐10xHis‐SUMO‐TEV‐RPAP3‐3C‐Strep‐II), and the same RPAP3 construct together with untagged PIH1D1 (NM_017916.2) (pRSFDuet1‐10xHis‐SUMO‐TEV‐RPAP3‐3C‐Strep‐II‐PIH1D1) were purchased from GenScript. Oligonucleotides used for cloning are shown in Table S1.

### Plasmid DNA and transient transfections

The pcDNA3.1 plasmids overexpressing WAC‐3xFlag or 3xFlag as control were generated in our laboratory by using the IVA cloning system [38]. pLJC5‐TMEM192‐2xFlag and pLJC5‐TMEM192‐mRFP‐3xHA (Addgene, Watertown, MA, USA) were the desired combination of cDNA constructs that were transiently transfected using the Lipofectamine 2000 method (Life Technologies, Waltham, MA, USA), following the manufacturer's instructions, for HEK293T cellular assays.

### Expression and purification of recombinant human WAC in Expi293 cells

C‐terminal 3xFlag‐tagged human WAC was overexpressed in Expi293 mammalian cells. The cells were lysed in WAC buffer A (50 mm Tris–HCl pH 8.0, 150 mm NaCl, 0.1% (v/v) NP‐40, 5% (v/v) glycerol) supplemented with benzonase (250 U·μL^−1^) (Novagen (MERK), Darmstadt, Germany) and cOmplete™ EDTA‐free protease Inhibitor Cocktail (Roche, Basel, Switzerland) by sonication and clarified by centrifugation at 50 000 ***g*** during 1 h at 4 °C. Protein purification was performed by incubating the clarified lysate with anti‐Flag resin (Anti‐DYKDDDDK G1 Affinity resin, Genescript) pre‐equilibrated in WAC buffer A for 2 h at 4 °C or 1 h RT. Resin was extensively washed with WAC buffer A and five times with WAC buffer B (100 mm Tris–HCl pH 8.0, 300 mm NaCl and 5% (v/v) glycerol). WAC protein was eluted by incubation for 30 min at 4 °C with WAC buffer B supplemented with 3xFlag peptide (MDYKDHDGDYKDHDIDYKDDDDK, QYAOBIO (ChinaPeptides), Shangai, China) (final concentration 0.3 mg·mL^−1^). Protein fractions were frozen in liquid N_2_and stored at −80 °C.

### Expression and purification of recombinant proteins in insect cells

#### Human mTOR‐mLST8

A recombinant baculoviral genome (bacmid) of the mTOR‐mLST8 complex with an N‐terminal 10xHis tag in mTOR was first generated in DH10EMBacY *E. coli* cells (Multibac Expression System, Geneva Biotech, Pregny‐Chambésy, Switzerland). Recombinant baculoviruses were produced by transfection of the bacmid into Sf9 insect cells using FuGENE® HD Transfection Reagent (Promega Corporation, Madison, WI, USA). Sf9 insect cells were infected for 72 h, and mTOR‐mLST8 overexpressing cells were lysed in mTOR‐mLST8 buffer A containing 50 mm HEPES pH 7.5, 150 mm NaCl, 1 mm EDTA, 0.5 mm TCEP, supplemented with benzonase (250 U·μL^−1^) (Novagen) and cOmplete™ EDTA‐free protease Inhibitor Cocktail (Roche). After sonication, the lysate was clarified by centrifugation at 50 000 ***g*** and 4 °C. The soluble fraction was incubated for 30 min with Ni‐NTA agarose beads (QIAGEN N.V. (Global Corporate Headquarters), The Netherlands) pre‐equilibrated in mTOR‐mLST8 buffer A supplemented with 20 mm imidazole and washed with mTOR‐mLST8 buffer B (50 mm HEPES pH 7.5, 300 mm NaCl, 1 mm EDTA, 0.5 mm TCEP, and 70 mm imidazole). The complex was eluted in mTOR‐mLST8 elution buffer (50 mm HEPES pH 7.5, 300 mm NaCl, 1 mm EDTA, 0.5 mm TCEP, and 500 mm Imidazole). Purified protein was dialyzed in 50 mm HEPES pH 7.5, 300 mm NaCl, 1 mm EDTA, and 0.5 mm TCEP buffer.

#### Human TELO2

TELO2 with N‐terminal GST tag followed by TEV protease site was produced in Sf9 cells using a similar protocol as described for mTOR‐mLST8. Cell lysis was carried out using a Dounce homogenizer in hypotonic buffer (100 mm Tris–HCl pH 8, 10 mm NaCl, 5 mm ß‐mercaptoethanol, 5% (v/v) glycerol, 0.1% (v/v) NP‐40) supplemented with cOmplete™ EDTA‐free protease Inhibitor Cocktail (Roche) and phosphatase inhibitors (PhosSTOP™, Roche). Lysate was supplemented with a final concentration of 300 mm NaCl, sonicated, and incubated with benzonase (125 U·μL^−1^) (Novagen) for 20 min at 4 °C. After clarification at 75 600 *g* and 4 °C for 1 h, the supernatant was incubated with Glutathione Sepharose 4B (Global Life Sciences Solutions (Cytiva), Wilmington, DE, USA) resin equilibrated in TTT buffer (100 mm Tris pH 8, 300 mm NaCl, 5 mm ß‐mercaptoethanol, 5% (v/v) glycerol) for 4 h at 4 °C with rocking. After extensive washing with TTT buffer, elution was performed by incubation for 30 min with TTT buffer supplemented with 50 mm l‐Glutathione reduced (Sigma‐Aldrich, St. Louis, MO, USA). Purified protein was digested with TEV protease for 16 h at 4 °C while dialyzing in TTT buffer supplemented with 1 mm DTT and 0.5 mm EDTA to remove the GST tag and further purified with Glutathione Sepharose 4B beads. Purified TELO2 was concentrated using an Amicon® Ultra‐4 50 K device (Millipore, Billerica, MA, USA).

#### Human TTI1‐TTI2

TTI1 containing a Twin‐Strep and a 3xHA tag followed by HRV 3C protease site was co‐expressed with untagged TTI2 using the same protocol described for TELO2. The lysate was incubated with BioLock Biotin Blocking Solution (IBA) prior to centrifugation, following the manufacturer's instructions. Clarified lysate was incubated with StrepTactin™XT Superflow™ resin (IBA Lifesciences, Göttingen, Germany) equilibrated in TTT buffer for 3 h at 4 °C and eluted with the same buffer supplemented with 50 mm Biotin (IBA Lifesciences).

### Expression and purification of recombinant proteins in bacteria

#### Human RUVBL1‐RUVBL2

10xHis‐RUVBL1‐RUVBL2 complex was produced as before [39]. Briefly, human RUVBL1 containing an N‐terminal His‐tag and TEV protease site and untagged RUVBL2 were co‐expressed in *E. coli* BL21 (DE3) cells, and the His‐RUVBL1‐RUVBL2 complex was purified by affinity chromatography using a HisTrap HP column (Cytiva). Untagged RUVBL1‐RUVBL2 complex was purified by digestion with TEV protease during 16 h at 4 °C and a second affinity purification step with HisTrap HP column (Cytiva).

#### Human RPAP3

N‐terminal 10xHis‐SUMO‐ and C‐terminal Strep‐tagged RPAP3 was expressed in *E. coli* BL21 Gold (DE3) cells by induction with 0.5 mm IPTG for 4 h at 28 °C. Cells were collected by centrifugation at 3990 *g* for 1 h and 4 °C, and lysed in R2TP lysis buffer (25 mm HEPES pH 7.8, 500 mm NaCl, 10% (v/v) glycerol, 0.5% (v/v) NP‐40) supplemented with 0.3 mg·mL^−1^ lysozyme, benzonase (250 U·μL^−1^) (Novagen) and cOmplete™ EDTA‐free protease Inhibitor Cocktail (Roche) by sonication after 20‐min incubation on ice. After centrifugation at 50 000 ***g*** for 1 h at 4 °C, the soluble fraction was incubated with StrepTactin™XT Superflow™ resin (IBA Lifesciences), pre‐equilibrated in R2TP buffer (25 mm HEPES pH 7.8, 300 mm NaCl, and 1 mm DTT) for 2 h at 4 °C. Unbound proteins were washed with R2TP buffer, and RPAP3 was eluted using the same buffer supplemented with 50 mm Biotin (IBA Lifesciences). The purified RPAP3 was subjected to a second step of purification by incubation for 30 min at 4 °C with Ni‐NTA agarose beads (Qiagen) equilibrated in R2TP buffer supplemented with 50 mm imidazole and without DTT. After extensive washing, the protein was eluted with R2TP buffer supplemented with 500 mm imidazole (and no DTT) and further dialyzed in R2TP buffer prior to storage.

#### Human RPAP3‐PIH1D1 complex

The RPAP3‐PIH1D1 heterodimer was produced by co‐expression of the 10xHis‐SUMO‐RPAP3‐Strep construct and untagged PIH1D1 in *E. coli* BL21 Gold (DE3) similar to RPAP3 and purified using the same protocol described for RPAP3.

#### Human R2TP complex

Expression of the R2TP complex (RUVBL1‐RUVBL2‐RPAP3‐PIH1D1) was performed by cotransformation in *E. coli* BL21 (DE3) cells with pETEV15b‐RUVBL1, pCDFDuet‐1‐RUVBL2, and pRSFDuet1‐10xHis‐SUMO‐TEV‐RPAP3‐3C‐Strep‐II‐PIH1D1 plasmids. Co‐expression was induced by adding 0.25 mm IPTG final concentration for 4 h at 28 °C. The R2TP complex was purified following the same protocol described above for RPAP3.

### Cell lines

Human embryonic kidney 293T (HEK293T; RRID:CVCL_0063) cells were kindly provided by Dr Hector Peinado (CNIO, Spain). The identity of the cell line was authenticated within the past 3 years by short tandem repeat (STR) profiling, performed by the CNIO's Monoclonal Antibodies Unit. All experiments were carried out using cells confirmed to be free of mycoplasma contamination, monthly tested using qPCR by the CNIO's Monoclonal Antibodies Unit. RagB and RagB‐Q99L overexpressing HEK293T cells were a kind gift from Dr Alejo Efeyan (CNIO, Spain). Cells were maintained in Dulbecco's modified Eagle's medium (DMEM) (Merck Life Sciences), supplemented with 10% (v/v) fetal bovine serum (FBS) (Thermo Fisher Scientific, Waltham, MA, USA) and sodium pyruvate 1 mg·L^−1^ (Merck Life Sciences, Darmstadt, Germany). Cells were tested for mycoplasma contamination and were maintained at 37 °C in a humidified 5% CO_2_ atmosphere. Sf9 (*Spodoptera frugiperda* 9) insect cells were cultured in suspension in Expression Systems' ESF 921™ Insect Cell Culture Medium (ESF921, Oxford Biomedical Research) and maintained at 27 °C. Expi293 mammalian cells were grown in suspension in Expi293™ Expression Media (Life Technologies, S.A.) and maintained at 37 °C with 5% CO_2_.

### Cell treatments

To perform glucose and glutamine modulation experiments, subconfluent cell cultures were rinsed twice and incubated in RPMI 1640 medium (US Biological) without amino acids and glucose, supplemented with 10% dialyzed fetal bovine serum (dFBS, amino acid free) and all 20 amino acids, except glutamine, during 3 h (see Table S2 for final concentrations of amino acids). Restimulation with glucose (Sigma‐Aldrich) and glutamine was performed for indicated times.

### Flag‐tag *in vitro* pull‐down experiments using purified proteins

Direct interactions of WAC with other proteins were tested using pull‐down experiments using WAC‐3xFlag overexpressed in Expi293 cells as bait. We purified WAC to homogeneity by affinity purification, as revealed by SDS/PAGE. However, in our hands, WAC was prone to aggregation and/or degradation over time and upon storage. We observed that these issues were overcome if eluting WAC from the beads following complex formation. Thus, the *in vitro* pull‐down experiments were designed to test interactions in the beads prior to elution.

For pull‐down experiments, WAC‐overexpressing cells were lysed in WAC buffer A2 (150 mm Tris–HCl pH 8.0, 200 mm KCl, 1% (v/v) NP‐40, 5% (v/v) glycerol, 0.5 mm TCEP), supplemented with benzonase (250 U·μL^−1^) (Novagen) and cOmplete™ EDTA‐free protease inhibitor cocktail (Roche) by using a Dounce homogenizer followed by sonication and clarified by centrifugation at 21 000 ***g*** during 1 h at 4 °C. Clarified lysate was incubated with anti‐Flag resin (Anti‐DYKDDDDK G1 Affinity resin, Genescript) pre‐equilibrated in WAC buffer A2 for 2 h at 4 °C or 1 h RT. Resin was extensively washed with WAC buffer B2 (150 mm Tris pH 8, 300 mm KCl, 10% (v/v) glycerol, 0.5 mm TCEP), followed by incubation for 30 min at 4 °C with buffer B2 supplemented with 5 mm ATP and 5 mm MgCl_2_, and washed five times with WAC buffer B2. Purified prey proteins or protein complexes were then added to the resin and incubated for 20 min at 4 °C. Unbound proteins were extensively washed with WAC buffer B2, and elution was performed by incubation for 30 min at 4 °C with WAC buffer B2 supplemented with 0.3 mg·mL^−1^ 3xFlag peptide (MDYKDHDGDYKDHDIDYKDDDDK, ChinaPeptides). In every experiment, three consecutive elutions were done. WAC protein complexes were analyzed by SDS/PAGE and western blot.

As a negative control, we used an Expi293 cells lysate where WAC‐3xFlag was not expressed (labeled as Expi293 lysate in figures). This allowed us to test simultaneously similar conditions while comparing results when WAC was expressed or not. As shown, none of the proteins analyzed eluted from the beads in the experiments where WAC‐3xFlag was not expressed, indicating no unspecific binding of the prey proteins to the resin.

### Immunoprecipitation assays

HEK293T cells were lysed in WAC buffer A 24–48 h after transfection. For Flag immunoprecipitation, cell lysates were cleared by centrifugation, and 5 mg was incubated for 2 h with 100 μL of agarose Flag resin (Genescript). Then, the resin was washed 5 times in WAC buffer A and five times in WAC buffer B. Elution was performed by using the 3xFlag peptide at 0.3 mg·mL^−1^, as previously indicated. Endogenous immunoprecipitation assays were performed by incubating 0.5 μg·mL^−1^ of mTOR antibody (Cell Signalling, Danvers, MA, USA), WAC antibody (Merck‐Millipore), or a control IgG (Millipore) with 5 mg of cell lysates in the presence of BSA (1 mg·mL^−1^) to block unspecific interactions, followed by re‐incubation with Recombinant Protein G‐Sepharose 4B (Life Technologies). The beads were washed 8–10 times with Triton IP Lysis Buffer (50 mm HEPES pH 7.5, 40 mm NaCl, 2 mm EDTA, 50 mm NaF, 1% (v/v) Triton X‐100, 10 mm Sodium Pyrophosphate), freshly supplemented with cOmplete™ EDTA‐free protease Inhibitor Cocktail (Roche) and PhosSTOP™ (Roche). In all endogenous protein immunoprecipitations, bound proteins were eluted with denaturing loading sample buffer and analyzed by western blotting.

### Generation of WAC knockout cells using CRISPR/Cas9 genome editing

The nucleotide guide sequences targeting human WAC were designed using the CRISPR design tool Breaking Cas (https://bioinfogp.cnb.csic.es/tools/breakingcas/). The guide sequences targeting exon 3 of human WAC are the following: #1 CATGAAAAGATGCGAGACGC, #2 TGACAGCACAGGTCACAGTA. The single guide RNAs (sgRNAs), tracrRNA (Integrated DNA Technologies, Iowa, USA), and the purified Cas9 that was a kind gift from Dr Rafael Fernández‐Leiro (CNIO, Spain) were transfected in HEK293T cells by following IDT instructions to form the RNP complex. 48 h post transfection, cells were seeded in 96‐well plate format with DMEM containing 10% (v/v) FBS. Single clones were expanded and screened for WAC by genomic DNA sequencing and Tracking of Indels by DEcomposition (TIDE) analysis (https://tide.nki.nl/) [40]. Genomic DNA (gDNA) was purified from clones, and the region surrounding the guide RNA targeting region was amplified with Q5 high‐fidelity DNA polymerase (New England Biolabs, Ipswich, MA, USA) using the following primers:

Forward: 5′‐GTGTGATTTGTTATCAGGAGTCTTATTGTAACGC‐3′.

Reverse: 5′‐CTTCTTCAAGCTAGAAATACTACTGCCGC‐3′.

### Generation of TMEM192‐control and TMEM192‐Lyso stable cell lines

For the generation of WT and WAC KO HEK293T cells stably expressing pLJC5‐TMEM192‐2xFlag (Control cells) and pLJC5‐TMEM192‐mRFP‐3xHA (HA‐Lyso cells), first pLJC5‐TMEM192‐2xFlag and pLJC5‐TMEM192‐mRFP‐3xHA constructs were transfected together with dR8‐9.1 and VSV‐G in HEK‐293‐T packing cells, using Lipofectamine 2000 (Life Technologies), according to the manufacturer's instructions, for the generation of the lentiviral particles. 48 h after transfection, supernatant was collected, centrifuged (250 *g*, 5 min), and filtered with 0.22‐μm filters to eliminate cellular debris. In parallel, HEK293T WT and WAC KO cells were seeded in a six‐well plate a day before transduction. Then, a 2× polybrene mixture was prepared by adding 0.5 mL complete medium + polybrene stock solution (final concentration 8 μg·mL^−1^) to the cells together with 0.5 mL TMEM192‐Control or TMEM192‐HA‐Lyso virus supernatant, previously generated. Next, 24 h after transduction, 1 mL of regular medium was added to the cells without removing the virus supernatant. After 48–72 h, cells were checked for positive RFP signal by fluorescence microscopy. Control and HA‐Lyso cells (in both WT and WAC KO HEK293T) were maintained under 1.5 μg·mL^−1^ puromycin treatment.

### Lysosome immunoprecipitation

WT stably expressing Flag‐tagged (Control cells) or HA‐tagged TMEM192 (HA‐Lyso cells) were plated in 15‐cm plates. Two 15‐cm plates of each cell line were used in each experiment and were processed separately to ensure a rapid isolation of lysosomes. The protocol was adapted from [41] as follows. Cells were rinsed with KPBS buffer (136 mm KCl, 10 mm KH_2_PO_4_ pH 7.25) supplemented with cOmplete™ EDTA‐free protease Inhibitor Cocktail (Roche) and PhosSTOP™ (Roche). Pelleted cells were resuspended in 2 mL of freshly prepared and cold KPBS buffer and homogenized 30 times, being consistent with the strength between samples. 50 μL of sample was preserved as whole cell extract (WCE). After centrifugation (1000 ***g***, 2 min, 4 °C), 50 μL of the supernatant containing different organelles, including lysosomes, were preserved as postnuclear supernatant (PNS) and the rest (~ 1.5 mL) was incubated with 200 μL prewashed (KPBS buffer) magnetic HA beads (Thermo Fisher Scientific) during 45 min (4 °C, 0.2 *g* rotation). After the binding, the flow through (FT) was conserved and HA beads were washed eight times with KPBS buffer. Then, elution was performed in Urea Buffer 8 m pH 7 at 25 °C. Lyso‐IP samples were analyzed by western blot analysis by loading 10% of the eluted volume and 0.1% of WCE, PNS, and FT.

### SDS/PAGE and immunoblot analysis

Protein concentration was measured using the Protein Assay Dye Reagent Concentrate (Bio‐Rad). Protein lysates were separated on 4–15% or 7.5% MINI‐PROTEAN TGX™ Precast Protein Gels (Bio‐Rad, Hercules, CA, USA), transferred onto nitrocellulose membrane (Amersham Protan Premium 0.45, GE Healthcare Life Sciences, Pittsburgh, PA, USA), and blocked for 1 h in 5% (w/v) BSA in TBS buffer. After incubation with primary antibodies overnight (o/n) and secondary antibodies (1 h, RT), the membranes were washed and analyzed using the LI‐COR Odyssey Infra‐red Imaging System. Immunoreactivity bands were measured with the image j software or by the software included in the Odyssey Infra‐red Imaging System. Primary antibodies used in western blotting with dilutions were as follows: monoclonal Anti‐FLAG M2 antibody (Sigma‐Aldrich, F1804, 1 : 2000), HA (Abcam #1091591; 1 : 1000), mLST8_GBL 86B8 (Cell Signaling #3274; 1 : 1000), mTOR (7C10, Cell signaling #2972; 1 : 1000), RagA/B D8B5 (Cell Signaling #4357; 1 : 1000), RAPTOR (24C12) (Cell signaling, #2280; 1 : 1000), S6 ribosomal protein (Cell Signaling #2217; 1 : 1000), p‐S6 ribosomal protein (Ser240/244) (Cell Signaling #2215; 1 : 1000), TELO2 (15975‐1‐AP, Proteintech, Rosemont, IL, USA; 1 : 1000), TTI1 (A303‐451A, Bionova Scientific; 1 : 1000), TTI2 (A303‐476A, Bionova Scientific, Fremont, CA, USA; 1 : 500), RPAP3 (Invitrogen #PA5‐58335; 1 : 500), RUVBL1 (Cell signaling #12300; 1 : 500), RUVBL2 (Cell signaling #8959; 1 : 500), PIH1D1 (Invitrogen PA5‐61482, 1 : 500). Secondary antibodies used were as follows: Anti‐Rabbit IgG (H + L) (DyLightTM 680 Conjugate) (Cell signaling #5366; 1 : 15 000) and Anti‐Mouse IgG (H + L) (DyLightTM 800 4X PEG Conjugate) (Cell signaling #5257; 1 : 15 000).

### Quantification of western blot images

Immunoreactivity bands of western blot were quantified by laser densitometry with the image j software (NIH) (https://imagej.nih.gov/ij/index.html) or by the software included in the Odyssey Infra‐red Imaging System.

### Immunofluorescence

Cells previously seeded in glass coverslips, precoated with gelatin 0.1% (Sigma‐Aldrich), were fixed in 4% paraformaldehyde (Thermo Fisher Scientific) (10 min). Permeabilization was carried out with 0.5% (v/v) Triton X‐100 in PBS (10 min), followed by blocking with 1% (w/v) BSA, 0.1% (v/v) Tween 20 in PBS for 1 h RT. The coverslips were incubated with primary antibodies in 1% BSA PBS (Flag 1 : 500 2 h RT), washed three times with PBS, and incubated with secondary antibodies for 45 min at RT. Cells were mounted with Fluoromount‐G (Southern Biotechnology Associates, Inc, Birmingham, AL, USA). Cell nuclei were counterstained with DAPI (Sigma‐Aldrich). Sample images were acquired using a confocal laser microscope SP5 WLL (Leica) at ×63 magnification. Secondary antibodies: Fluor 488 Goat Anti‐Rabbit IgG (H + L), Fluor 555 Goat Anti‐Rat IgG (H + L), Fluor 647 Goat Anti‐Mouse IgG (H + L) (1:500) (Life Technologies). image j software (NIH) was used for general image analysis.

### Transcriptomic and proteomic analysis

We analyzed the gene and protein levels of the WAC complex using comprehensive proteogenomic datasets from the Clinical Proteomic Tumor Analysis Consortium (CPTAC), which includes data from over 1000 primary tumors across 10 cancer types [42]. Transcriptomic and proteomic data were retrieved from the LinkedOmicsKB data portal (https://kb.linkedomics.org/), comprising normalized RNA sequencing and log₂‐transformed MS1 intensity values for protein abundance from tumor and matched normal samples. The MS1 intensity reflects precursor ion signal measured during mass spectrometry, and the log₂ transformation was applied by the original data provider to normalize dynamic range. Z‐score normalization was performed for each gene and protein by subtracting the overall mean across cancer types and dividing by the standard deviation. Statistical analyses were performed in r (v.4.4.1) using stringr (v.1.5.1), tidyr (v.1.3.1), dplyr (v.1.1.4), purr (v.1.0.2), tibble (v.3.2.1), and stats (v.4.4.1), and figures were generated using ggplot2 (v.3.5.1) and pheatmap (v.1.0.12).

### Statistics

All data are presented as mean values ± SEM of the indicated number of independent experiments stated in the figure legend. We determined the statistical significance in instances of single comparisons by unpaired Student's *t* test or two‐way ANOVA as indicated. Statistics were obtained using the graphpad prism 8 software (https://www.graphpad.com/). The differences were considered significant when *P < 0.05, **P < 0.01, ***P < 0.001.

## Results

### WAC directly interacts with mTOR and TELO2 but not TTI1 and TTI2

Previous work by David‐Morrison *et al*. showed by immunoprecipitation that WAC interacts with mTOR, RAPTOR, RUVBL1, RUVBL2, TTI1, and TELO2 in cells [15]. We immunoprecipitated endogenous WAC in HEK293T cells and used mass spectrometry analysis to identify interactors. WAC associates with mTOR, RAPTOR, and mLST8 (Fig. S1). Endogenous WAC also precipitated TELO2 and TTI1 (components of the TTT complex), and RUVBL1 and RUVBL2, components of R2TP. These results are consistent with the results obtained by David‐Morrison *et al*. [15]. Interestingly, we did not detect TTI2, the third partner in the TTT complex, nor PIH1D1 and RPAP3, despite observing a direct interaction with WAC *in vitro* (see below). WAC recognizes most of the components of the TTT and R2TP complexes (see below), but it is likely that these interactions take place in the context of different complexes in cells, maybe involving mutually exclusive interactions. Also, some interactions could be weaker than others, and this variation may influence their detection depending on the experimental conditions and techniques used. In support of this possibility, David‐Morrison *et al*. were unable to co‐immunoprecipitate mTORC1, R2TP, and TTT in HEK293T cells using a WAC antibody unless a cross‐linking agent was used, and they suggested that these interactions were weak or transient [15]. However, they were able to detect stronger interactions without the use of a cross‐linker when cells were first starved and subsequently fed with glucose and glutamine, supporting that these interactions could be affected by the experimental conditions.

Immunoprecipitation experiments in cells cannot distinguish between proteins that interact directly with WAC and those associated with it indirectly as part of a larger multi‐subunit complex. Characterizing the proteins that interact directly with WAC will contribute to understanding how WAC regulates TTT‐R2TP and mTORC1 activity. To address this, we first purified proteins obtained after recombinant expression and affinity purification (Fig. S2). mTOR was produced in complex with mLST8 for its stability. For the TTT complex, TTI1‐TTI2 have been shown to form a stable subcomplex on their own where TELO2 associates [11], and therefore we purified TELO2 and TTI1‐TTI2 separately.

**Fig. 1:**
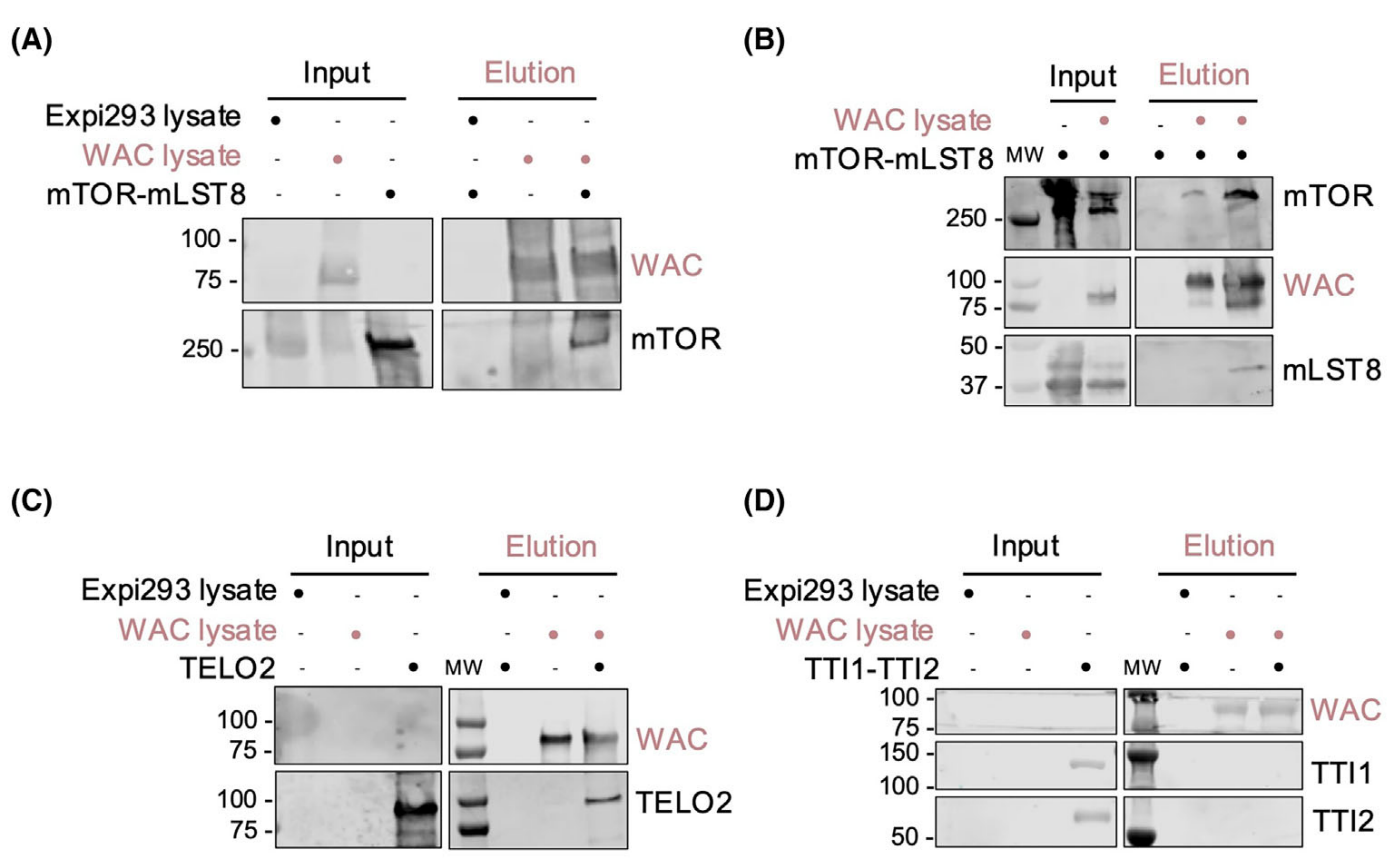
WAC directly interacts with mTOR and TELO2. (A) Pull‐down experiment testing the interaction between WAC‐3xFlag and purified mTOR‐mLST8. Western blot of the co‐eluted proteins was only performed against the mTOR subunit. (B) WAC interacts directly with mTOR‐mLST8. Immunoblot analysis of the interaction of WAC with mTOR and mLST8 after elution of WAC from an affinity chromatography from lysates of HEK293 cells co‐expressing WAC‐3xFlag and mTOR‐mLST8. Two consecutive elutions are shown for the WAC + mTOR‐mLST8 sample. (C) Same experiment as in (A) but testing the interaction of WAC with TELO2. (D) Interaction of WAC and TTI‐TTI2 as in (C). Inputs and elutions were analyzed by SDS/PAGE and western blot. Inputs show the soluble fraction of Expi293 cell lysates expressing either 3xFlag as a control (labeled as Expi293 lysate) or WAC‐3xFlag (labeled as WAC lysate).

We designed an optimized experimental setup to test the direct interaction of WAC with mTOR, TELO2, and TTI‐TTI2 by immobilizing purified WAC on beads, coupling the purification step with the interaction pull‐down experiment. For this, we purified WAC‐3xFlag overexpressed in Expi293 cells with anti‐FLAG agarose resin, which was extensively washed to remove any contaminant protein besides WAC. Then, the purified WAC loaded on the beads was incubated with each of the purified proteins separately, further washed, and eluted. WAC and the co‐eluting proteins were identified by western blot (Fig. 1). As a control in all these experiments, we used a lysate of Expi293 cells where WAC‐3xFlag was not expressed, which was incubated with the anti‐FLAG beads and then subjected to the incubation with the prey proteins. Also, we included in each experiment an assay of Expi293 cells overexpressing WAC‐3xFlag that was not incubated with any partner, as a control of WAC expression.

We found that WAC directly interacted with the mTOR‐mLST8 complex (Fig. 1A). A similar result was obtained by co‐expressing WAC‐3xFlag and mTOR‐mLST8 in HEK293T, followed by a purification using WAC as bait, where we observed mTOR and mLST8 co‐eluting with WAC (Fig. 1B). WAC also interacted with the TELO2 subunit of the TTT complex when analyzed in pairwise interactions (Fig. 1C). However, WAC did not interact with the subcomplex formed by TTI1 and TTI2 (Fig. 1D).

Together, these results indicate that WAC interacts directly with mTOR and TELO2, but not with TTI1‐TTI2, at least under the conditions tested.

### WAC interacts with the R2TP complex

The R2TP complex is formed by the interaction between two subcomplexes, RUVBL1‐RUVBL2 and RPAP3‐PIH1D1 [1, 2]. Thus, we purified RUVBL1‐RUVBL2 and the RPAP3‐PIH1D1 heterodimer as two separate subcomplexes to test their *in vitro* interaction with WAC (Fig. S2). The full R2TP was produced and purified to homogeneity after co‐expression of the four subunits and affinity purification using RPAP3 as bait, efficiently recovering all the components of the complex (Fig. S2).

**Fig. 2:**
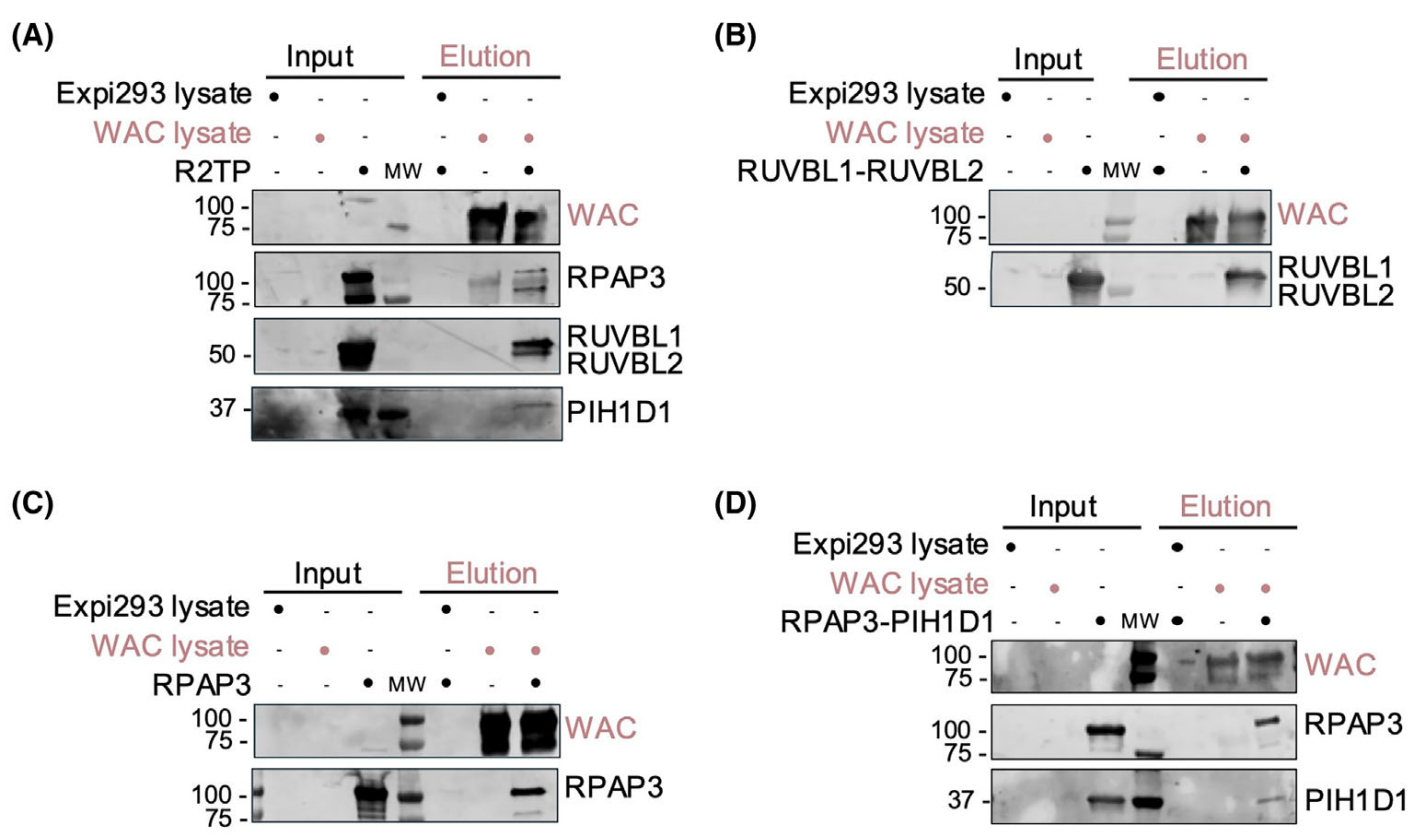
WAC interacts with the R2TP complex. (A) Pull‐down experiment testing the interaction between WAC‐3xFlag and the R2TP complex. WAC‐3xFlag, used as bait, was incubated with a purified, already assembled R2TP complex. (B) To map the interaction of WAC with the subunits of the R2TP complex, a similar experiment as in (A) was performed to test the interaction with the RUVBL1‐RUVBL2 complex. Similar experiments are shown but using RPAP3 (C) and RPAP3‐PIH1D1 (D) as prey. Assays were performed as explained in the Methods section, similarly to the experiments shown in Fig. 3C–E.

Using the protocol described above, we studied the direct binding of WAC with purified R2TP complex (Fig. 2A). In these experiments, WAC co‐eluted with all four components of the complex, RUVBL1‐RUVBL2, RPAP3, and PIH1D1. Furthermore, WAC also interacted with the RUVBL1‐RUVBL2 module, with the RPAP3‐PIH1D1 heterodimer, and with the RPAP3 subunit when each of these subcomplexes was tested separately (Fig. 2B–D). We were unable to test the direct interaction between WAC and PIH1D1 because, whereas RPAP3 can be produced on its own, PIH1D1 could only be produced as a complex with RPAP3 as it tended to aggregate in our hands [1, 2]. Thus, these results show that WAC has the capacity to directly interact with components of both subcomplexes of the R2TP: RPAP3 on one hand, and the RUVBL1‐RUVBL2 ATPase module on the other hand. In this latter case, we cannot discern whether the interaction of WAC is through RUVBL1, RUVBL2, or both.

### Correlated overexpression of WAC, R2TP, and TTT in diverse cancer types

mTOR is frequently found to be hyperactivated in human cancers, and the mechanisms behind its regulation are of great interest for the development of new treatments [43]. The interaction of WAC with mTORC1 and with the R2TP‐TTT chaperone suggests that there should also be functional links among these proteins that could reflect in a correlated transcription and/or protein expression in some cancer contexts. Thus, we explored the biological relevance of the interactions between WAC, mTOR, and the R2TP‐TTT chaperone system by analyzing both transcriptomic and proteomic data from CPTAC (The National Cancer Institute's Clinical Proteomic Tumor Analysis Consortium) database, which integrates cancer proteogenomic data of more than 1000 treatment‐naive primary tumors from 10 cancer types [44].

**Fig. 3:**
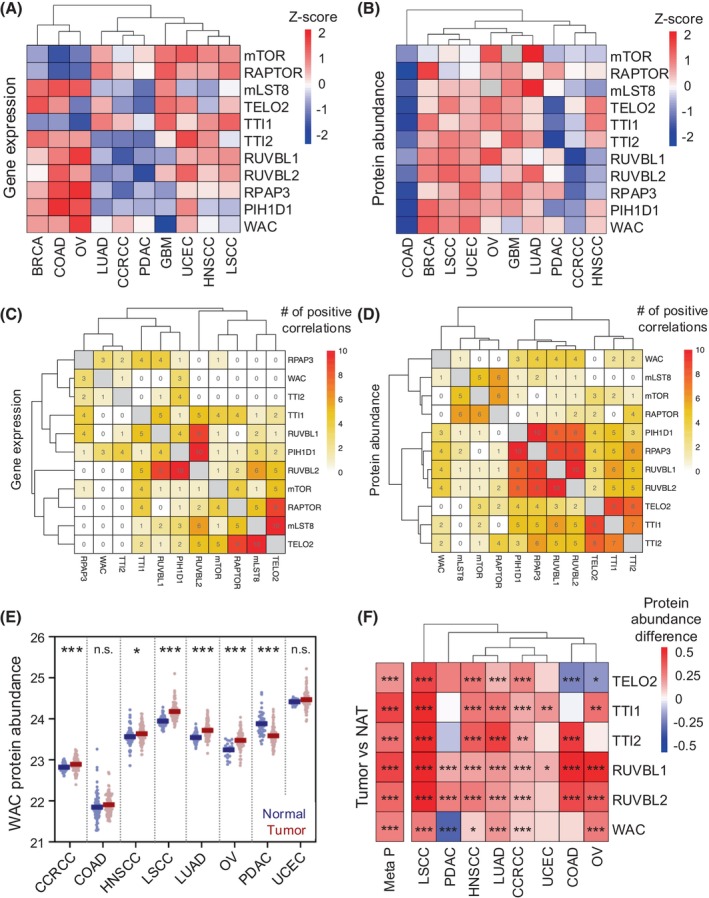
Analysis of gene and protein levels for WAC, mTORC1, R2TP, and TTT across cancer types. Gene (A) and protein (B) levels of the subunits of the mTOR‐WAC‐RUVBL1‐RUVBL2‐TTT complex in 10 cancer types with CPTAC tumor samples from LinkedOmics. Z‐scores are shown to represent normalized expression levels, with red indicating relatively high expression and blue indicating relatively low expression. The number of cancer types showing a positive correlation in gene (C) and protein (D) levels between subunits of the mTOR‐WAC‐RUVBL1‐RUVBL2‐TTT complex across 10 cancer types. The number within each box indicates the number of cancer types with a positive correlation out of the 10 tested. A positive correlation is defined as a Pearson correlation coefficient > 0.2 with a *P*‐value < 0.01. (E) Distribution of WAC protein expression in tumor samples versus matched normal samples across cancer types (where normal samples are available). Statistically significant differences between tumor and normal conditions were assessed using the two‐sided Wilcoxon rank sum test. Units in the *Y* axis correspond to protein expression levels in LinkedOmics, provided as log₂‐transformed MS1 intensities, which represent relative protein abundances measured via mass spectrometry and normalized for comparative analyses. (F) Differences in protein levels of the components of the WAC‐RUVBL1‐RUVBL2‐TTT complex between tumor and adjacent normal tissue (NAT) across cancer types. Red indicates higher expression in tumors compared to normal samples, while blue indicates higher expression in normal samples compared to tumors. Statistically significant differences were assessed using the two‐sided Wilcoxon rank sum test. The meta *P*‐value represents pan‐cancer significance when all samples are analyzed together using the two‐sided Wilcoxon rank sum test. *P < 0.05, **P < 0.01, ***P < 0.001. BRCA, breast invasive carcinoma; CCRCC, clear cell renal cell carcinoma; COAD, colon adenocarcinoma; GBM, glioblastoma; HNSCC, head and neck squamous cell carcinoma; LSCC, lung squamous cell carcinoma; LUAD, lung adenocarcinoma; OV, ovarian serous cystadenocarcinoma; PDAC, pancreatic ductal adenocarcinoma; UCEC, uterine corpus endometrial carcinoma.

We first characterized cancer‐type‐specific variations in the expression of WAC, mTORC1, and R2TP‐TTT components, followed by an analysis of potential co‐expression patterns. To minimize baseline variability across cancer types, transcriptomics and proteomics data were independently Z‐score normalized, where expression levels were mean‐centered and scaled by the standard deviation (Fig. 3A,B; Fig. S3A,B). This allowed us to compare relative expression patterns across cancer types while minimizing baseline variability. We observed that WAC and components of mTORC1, R2TP, and TTT complexes are broadly expressed at both mRNA and protein levels across multiple tumor types (Fig. 3A,B). Expression varies across tumor types, which may reflect a more prominent involvement of these proteins in certain cancers such as breast, lung, or ovarian carcinomas, as reported in previous studies [33].

Correlation analysis of mRNA levels across cancer types was then performed to assess associations among the components. Strong associations were revealed between RUVBL1 and RUVBL2, which was expected as they are known to form a complex. But in addition, we also observed a strong correlation between TELO2 and RAPTOR (in nine out of 10 cancer types), mLST8 (in 10 out of 10 cancer types), mTOR, and RUVBL2 (in five out of 10 cancer types) (Fig. 3C). WAC expression was also correlated with that of RPAP3 and PIH1D1 (in three out of 10 cancer types), components of R2TP, and we also found some correlation with TTI2. Given that these proteins physically interact in cells, their consistent co‐expression across tumor types may reflect an underlying regulatory coordination, potentially related to mTOR pathway modulation.

Positive correlation analysis in protein expression can recapitulate interactions as part of macromolecular complexes as well as interactions as part of functional pathways, and identify correlations absent at the mRNA level [45]. We thus analyzed correlations at the level of protein expression. We detected stronger associations between the four components of R2TP and between the three components of TTT (in 8 to 10 out of 10 cancer types), which supports that this analysis can serve to identify functional interactions (Fig. 3D). In addition, we also detected associations between WAC and components of R2TP (three to four out of 10 cancer types) and TTT (2 out of 10 cancer types) (Fig. 3D), which is consistent with the involvement of WAC, R2TP, and TTT in the same pathway. WAC expression was also correlated with mLST8, supporting a connection of WAC with mTORC1.

Finally, we analyzed whether there could be changes in the protein expression of WAC in some tumors. Analysis of WAC protein levels revealed that these were significantly upregulated in most cancer types (five out of eight; *P*‐value < 0.05), including lung squamous cell carcinoma (LUSC) and ovarian serous cystadenocarcinoma (OV) (Fig. 3E). In contrast, WAC protein levels were reduced in pancreatic ductal adenocarcinoma, suggesting that its regulation may be tumor‐type specific (Fig. 3E). We then expanded the same analysis to the components of the R2TP and TTT complexes, observing a widespread and significantly higher protein abundance of all these proteins in tumors compared with matched normal tissues (Fig. 3F).

Together, all these results reveal that the expression of WAC is correlated with components of the R2TP, TTT, and mTORC1 complexes in several tumors, reinforcing the notion that WAC and the R2TP‐TTT chaperone complexes functionally cooperate to regulate mTORC1 in cancer.

### WAC preferentially interacts with mTOR and the TTT complex under energetic stress

**Fig. 4:**
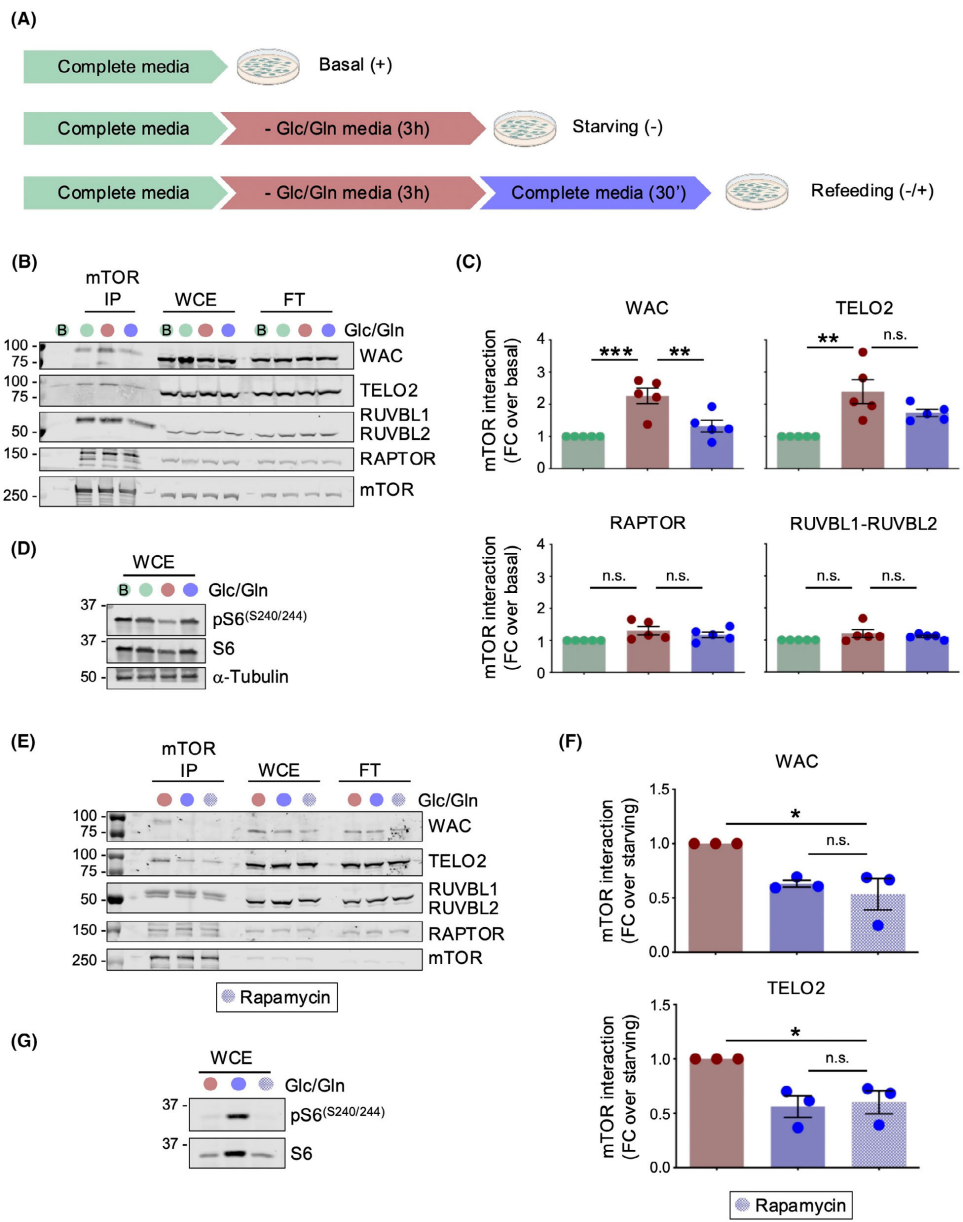
WAC preferentially interacts with mTOR and the TTT complex in the absence of glucose and glutamine. (A) Schematic illustration of nutrient stress protocol used. Color codes for each condition are used for the rest of the panels in the fig. (B) Immunoblot analysis of endogenous mTOR immunoprecipitation (IP) assay performed in HEK293T under Glc/Gln modulation. Representative blot of 5 independent experiments is shown. Each lane is labeled with a color code corresponding to the conditions indicated in (A). A control experiment, indicated as a B within a green circle, was performed using only beads without antibody and using complete media. (C) Immunoprecipitation data (mean ± SEM) from (B) were normalized by total mTOR immunoprecipitated and expressed as fold change of association with respect to the basal condition. Statistical significance was analyzed using ordinary one‐way ANOVA and Tukey's multiple comparisons test. *P*‐values, *P < 0.05, **P < 0.01, ***P < 0.001. (D) Immunoblot analysis of phosphorylation status of S6 is shown as a control of mTORC1 modulation in (B) and (C). (E) Immunoblot analysis of endogenous mTOR performed as in (B), treating cells with rapamycin for 30 min prior to Glc/Gln recovery. Representative blot of three independent experiments is shown. Color used for conditions using rapamycin is shown at the bottom and used in other panels. (F) Immunoprecipitation data (mean ± SEM) from (E) were normalized by total mTOR immunoprecipitated and expressed as fold change of association with respect to the starving condition. Statistical significance was analyzed using ordinary one‐way ANOVA and Tukey's multiple comparisons test. *P*‐values, *P < 0.05. (G) Immunoblot analysis of phosphorylation status of S6 is shown as a control of mTORC1 modulation in (E) and (F).

Based on the hypothesis that WAC regulates mTORC1 through R2TP and TTT chaperone complexes in a nutrient‐sensitive manner, we assessed whether the interactions of mTOR with WAC and these chaperones change under basal, starvation, and refeeding conditions. We immunoprecipitated endogenous mTOR and analyzed its interaction with WAC, RUVBL1‐RUVBL2 (R2TP), TELO2 (TTT), and RAPTOR (mTORC1) in three conditions: complete media (basal), after simultaneous deprivation of both Glc/Gln (starving), and after refeeding (Fig. 4A). We detected RAPTOR co‐immunoprecipitating with mTOR, confirming the presence of mTORC1 (Fig. 4B). WAC, RUVBL1‐RUVBL2, and TELO2 were also associated with mTOR in the IP for the different conditions (Fig. 4B).

Notably, WAC and TELO2 significantly increased their association with mTOR in Glc/deprivation conditions and dissociated after replenishment (Fig. 4C). TTI1 and TTI2, the partners of TELO2 in the TTT complex, also associated with mTOR more strongly during starving conditions than after refeeding (Fig. S4). The nutrient‐sensitive interaction of mTOR with WAC and TTT correlated with the inactivation of mTORC1 in starved conditions, measured by the phosphorylation levels of S6 ribosomal protein (Fig. 4D). The modulation in the activity of mTORC1 was not a result of changes in the association between endogenous mTOR and RAPTOR, which remained constant regardless of Glc/Gln levels (Fig. 4C). The association of mTOR with RUVBL1 and RUVBL2 also remained unaltered throughout the experiment (Fig. 4B,C). These results show that the interaction of mTOR with the components of the TTT‐R2TP chaperone WAC and TELO2 is sensitive to Glu/Gln deprivation‐replenishment.

mTOR is known to be inactivated upon Glc/Gln deprivation, and we wanted to test whether the kinase activity of mTOR could influence how mTOR associates with WAC, TELO2, and RUVBL1‐RUVBL2 after Glc/Gln addition. For this, the same experiment described above was repeated, but this time we compared cells treated and untreated with rapamycin for 30 min prior to Glc/Gln recovery, and we quantified the changes in WAC and TELO2 associated with mTOR with respect to the starving condition in each of the experiments (Fig. 4E–G, Fig. S4). We found no differences between the experiment when using rapamycin or not, suggesting that this association of mTOR with WAC and TELO2 does not depend on the kinase activity of mTOR. Also, we did not find changes for the association of mTOR with RUVBL1 and RUVBL2.

Together, these results reveal an enhanced association of WAC and the TTT complex with mTOR upon Glc/Gln depletion that correlates with a reduction in mTORC1 activity and their dissociation following nutrient replenishment. These changes seem to occur without affecting the interaction of mTOR with RAPTOR.

### WAC is not lysosome‐associated

mTORC1 relocates to the lysosomal surface for activation, and we wondered whether WAC could interact with mTOR at the lysosome where full activation occurs, and whether WAC localization could be altered with the nutrient conditions.

**Fig. 5:**
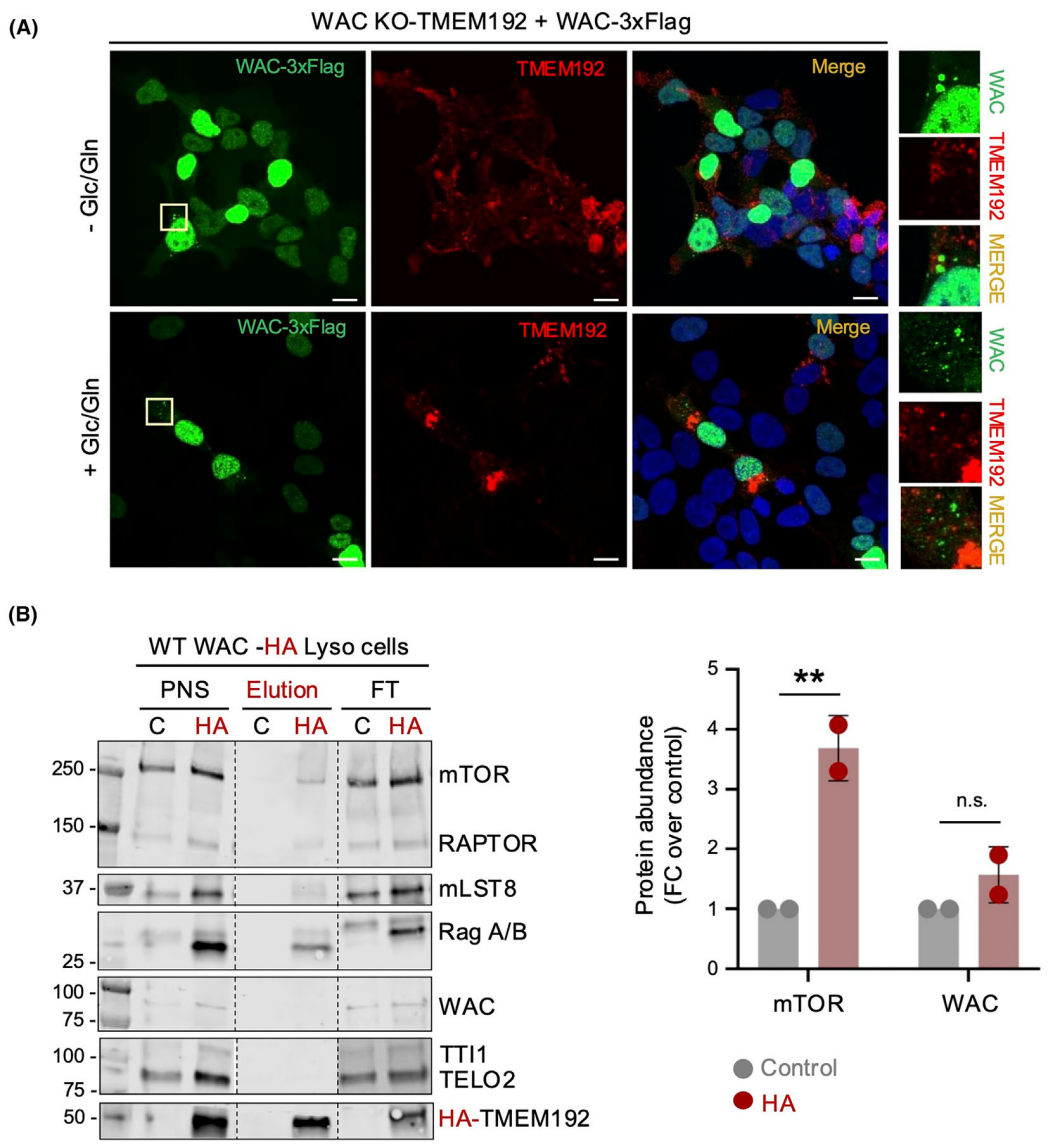
WAC does not localize at lysosomes. (A) WAC KO‐TMEM192 HA Lyso cells transiently overexpressing 3xFlag‐tagged WAC were stimulated with Glc/Gln for 30 min after starvation. Representative confocal micrographs (maximum intensity projections), before (top panels) and after (bottom panels) addition of Glc/Gln, showing signals of Flag (green), TMEM192 (red) and DAPI (blue). Scale bars, 10 μm. (B) Immunoblots of Lyso IP samples from control and TMEM192 HA Lyso WT HEK293T cells cultured under fed conditions. HA‐tagged TMEM192 was immunoprecipitated with HA beads, and IP and P post nuclear supernatant (PNS) were probed for the indicated proteins. Representative blot of two independent experiments. (C) Quantification of the experiment shown in (B), measuring the fold change (FC) in protein signal over the control. Statistical significance was analyzed using ordinary one‐way ANOVA and Tukey's multiple comparisons test. *P*‐values, **P < 0.005.

We first investigated the subcellular localization of WAC upon Glc/Gln depletion and recovery using confocal microscopy. WAC KO cells were generated by using CRISPR/Cas9 technology in HEK293T cells (Fig. S5) that stably overexpressed the lysosomal transmembrane protein 192 (TMEM19) (red), in which we re‐expressed WAC (green). WAC localized predominantly at the nucleus as previously described [34] and at the cytoplasm. WAC did not colocalize with lysosomes either in starved or refed conditions (Fig. 5A). To support this, we performed lysosome immunoprecipitation assays (Lyso‐IPs) in cells that stably expressed a HA‐tag version of the lysosomal protein TMEM192 [41] (Fig. 5B). mTORC1 components (mTOR, RAPTOR, and mLST8) and RagA/B GTPases co‐immunoprecipitated with lysosomes; however, WAC was not detected in the lysosomal fraction (Fig. 5B,C). The signal for WAC in the post nuclear supernatant (PNS) is weaker than for mTOR in these experiments, and we cannot completely discard that there could be minor amounts in the elution that could be undetected in these experiments. However, the signal for RAPTOR is also weak in PNS, but this is detected in the HA elution, and TELO2, a protein to which WAC interacts, is also clearly not detected in lysosomes in our experiment.

Together, these results show that WAC does not locate at detectable levels under our experimental conditions at the lysosomal surface, and we did not detect changes between starved and fed conditions. This suggests that the observed associations between WAC, R2TP, TTT, and mTOR likely occur with a cytosolic pool of mTORC1.

## Discussion

An active mTOR pathway plays an essential role in the regulation of cellular growth and metabolism. This pathway is aberrantly regulated in several diseases, including aging, obesity, and several cancers. Despite increased knowledge over the past two decades about the mechanisms regulating mTOR, the role of the TTT and R2TP chaperones in mTOR activation has only recently been appreciated [15, 32, 33]. The TTT complex acts as a chaperone that protects newly synthesized mTOR during its translation [12], which is then transferred to R2TP for the incorporation of RAPTOR to mTOR and mTORC1 dimerization [3, 4, 8, 9, 11]. This transfer is accomplished through the formation of a complex between TTT and R2TP, which involves the interaction of TTI1‐TTI2 with RUVBL1‐RUVBL2 [11].

David‐Morrison *et al*. demonstrated that WAC interacts with mTOR and with components of the R2TP and TTT chaperone complexes, and they proposed that WAC acts as an adaptor that helps in the interaction between TTT and RUVBL1‐RUVBL2 to facilitate mTORC1 assembly and activation [15]. WAC comprises multiple sites for tyrosine phosphorylation and participates in diverse complexes, including complexes with Polo‐like Kinase 1 (Plk1) [34] and with RNF20‐RNF40 [35], enabling the regulation of distinct cellular processes. However, our molecular understanding of how WAC interacts with mTOR, R2TP, and TTT remains poorly defined.

While co‐immunoprecipitation in cells cannot discriminate direct interactions from indirect associations within larger complexes, we mapped direct binders of WAC using purified proteins. We demonstrate that WAC interacts directly with mTOR‐mLST8, R2TP, and TELO2, but not with TTI1 nor TTI2, the other two components of the TTT complex. Interestingly, we find that in cells these interactions change between basal, starved, and refeeding conditions. We detected a more prominent interaction between WAC, mTOR, and TELO2 following deprivation of glucose and glutamine, and these interactions were weakened rapidly after nutrient refeeding. Interestingly, the formation and disassembly of the interactions between WAC, mTOR, and TELO2 correlated with changes in the activity of mTORC1. Together, these results suggest that the interaction of WAC with mTORC1 and the R2TP‐TTT chaperone system changes depending on nutrient conditions, and that these changes could serve to regulate mTORC1 activity. Our transcriptomic and proteomic analyses supported a relevant role of WAC in the R2TP‐TTT pathway by revealing that WAC expression and protein levels are correlated with those of R2TP and TTT in several tumors, suggesting that they are part of a functional pathway that contributes to some cancers.

Kim *et al*. found that glucose and glutamine deprivation downregulate mTORC1 by disrupting mTORC1 dimerization and lysosomal localization and that the mechanism involves the disruption of the interaction between TTT and RUVBL1‐RUVBL2 [33]. After removal of glucose and glutamine, we observe a strong interaction between WAC and TTT, whereas the levels of RUVBL1 and RUVBL2 associated with mTOR remain unaltered. Thus, it is conceivable that WAC could be the mediator linking energetic stress to the disruption of the TTT‐RUVBL1‐RUVBL2 complex, as observed by Kim *et al*. [33].

Our results revealed that a stronger association of WAC, mTOR, and TELO2 during starving was correlated with a lower activity of mTORC1. This may suggest that WAC could have some role in downregulating mTORC1. However, David‐Morrison *et al*. proposed a model where WAC would act as an adaptor for the TTT and RUVBL1‐RUVBL2 complexes to promote mTORC1 activation [15]. Our data cannot yet define whether WAC operates as an activator or inhibitor of mTORC1. Although we observe changes in the interactions between WAC, TELO2, and mTOR depending on the nutrient conditions, our experiments in cells and using purified proteins show that WAC interacts not only with TELO2 but also with RUVBL1‐RUVBL2 and R2TP. Since TTT and RUVBL1‐RUVBL2 need to interact between themselves to promote mTORC1 assembly [11], the interplay of WAC with all these proteins could be complex and imply mutually exclusive interactions and the assembly of different complexes. In addition, the interaction of WAC with other proteins, such as Plk1, is known to be regulated by the phosphorylation status of WAC [34]. This could add another layer of complexity in the regulation of the interaction of WAC with mTOR and TTT‐R2TP. Furthermore, WAC could also modulate the interaction of R2TP with other complexes, such as PFDL, since R2TP is known to interact with PFDL to form the PAQosome, a much larger assembly involved in the maturation of several macromolecular complexes [6, 14]. Therefore, our results clearly show that there is a dynamic interplay of interactions between WAC, R2TP, TTT, and mTOR depending on nutrient conditions, but how these changes are mechanistically linked to the regulation of mTORC1 remains to be explored.

Together, our results reveal insights on how WAC interacts with the TTT‐R2TP chaperone system to regulate mTORC1 activity in response to energetic stress. The R2TP complex controls the assembly and maturation not only of mTOR and other kinases of the PIKK family, but also of an ample collection of large macromolecular complexes [46]. It has already been proposed that accessory factors interact with R2TP and regulate specific functions or help as scaffolds between the chaperone machinery and specific clients [6]. Our results further characterize how the interaction of WAC with the R2TP and TTT chaperone complexes serves to regulate mTORC1 activation in response to metabolic stress.

## Conflict of interest

The authors declare no conflict of interest.

## Author contributions

SC designed and performed cell biology experiments; SC and CR organized all the data and prepared the first draft of the manuscript, including most figures; NC, AL‐P, CG‐M, AG‐C, and MS performed protein production and pull‐downs with purified proteins; AL‐P helped to prepare final versions of the figures; DM helped with the analyses of the experiments from confocal microscopy; AM‐R and SP performed all computational analyses based on cancer genomics; AE provided advice and revised the manuscript; OL designed the overall research and prepared the final version of the manuscript.

## Supporting information


**Fig. S1.** Mass spectrometry of WAC interactors in HEK293T cells using WAC antibodies.
**Fig. S2.** Purified proteins used in pull‐down experiments.
**Fig. S3.** Gene and protein expression analysis of WAC‐RUVBL1/2‐TTT complex components across cancer types.
**Fig. S4.** mTOR forms a complex with TTI1/TTI2 upon glucose and glutamine depletion.
**Fig. S5.** Characterization of WAC KO clones used in Fig. 2.
**Table S1.** Oligonucleotides used for cloning.
**Table S2.** Amino acids and glucose concentrations in RPMI 1640.

## Data Availability

This study re‐analyzed a LinkedOmicsKB, which was generated by The Clinical Proteomic Tumor Analysis Consortium (CPTAC, DOI: 10.1016/j.cels.2023.07.007). RNA expression data (RNAseq_gene_RSEM_coding_UQ_1500_log2_Tumor.txt; RNAseq_gene_RSEM_coding_UQ_1500_log2_Normal.txt) and protein expression data (proteomics_gene_abundance_log2_reference_intensity_normalized_Tumor.txt; proteomics_gene_abundance_log2_reference_intensity_normalized_Normal.txt) were downloaded from https://kb.linkedomics.org/download alongside clinical data of patients. Most other data underlying this article are available in the article and in its Supporting Information. All other data underlying this article not included in the article and Supporting Information will be shared upon request to the corresponding author. Code availability: Custom scripts constituting the pipeline used to analyze CPTAC consortium data are available from GitHub (https://github.com/SolipParkLab/WAC_bioinformatics).
