# Supplementary material for: Characterization of WAC interactions with R2TP and TTT chaperone complexes linking glucose and glutamine availability to mTORC1 activity
Source: FEBS Open Bio. 2025 Jul 13;15(11):1771–88. doi: 10.1002/2211-5463.70085 (PMC12582974; doi:10.1002/2211-5463.70085)
Supplement: Supplementary file 1 — Fig. S1. Mass spectrometry of WAC interactors in HEK293T cells using WAC antibodies. Fig. S2. Purified proteins used in pull‐down experiments. Fig. S3. Gene and protein expression analysis of WAC‐RUVBL1/2‐TTT complex components across cancer types. Fig. S4. mTOR forms a complex with TTI1/TTI2 upon glucose and glutamine depletion. Fig. S5. Characterization of WAC KO clones used in Fig. 2. Table S1. Oligonucleotides used for cloning. Table S2. Amino acids and glucose concentrations in RPMI 1640. [file FEB4-15-1771-s001.docx]

Supplementary Information for

**Characterization of WAC interactions with R2TP and TTT chaperone complexes linking glucose and glutamine availability to mTORC1 activity**

Sofía Cabezudo^1&^, Natalia Cuervo^1&^, Carmen García-Martín^1&^, Andrés López-Perrote^1^, Clara Reglero^1^, Adrián Maqueda-Real^1^, Ana González-Corpas^1^, Marina Serna^1^, Diego Megías^2,$^, Alejo Efeyan^3^, Solip Park^1^, Oscar Llorca^1*$^

1. Spanish National Cancer Research Centre (CNIO), Structural Biology Programme, Melchor Fernández Almagro 3, 28029 Madrid, Spain
2. Spanish National Cancer Research Centre (CNIO), Spanish National Cancer Research Centre (CNIO), Confocal Microscopy Unit, Melchor Fernández Almagro 3, 28029 Madrid, Spain
3. Spanish National Cancer Research Centre (CNIO), Molecular Oncology Programme, Melchor Fernández Almagro 3, 28029 Madrid, Spain

**Supplementary figures**

**
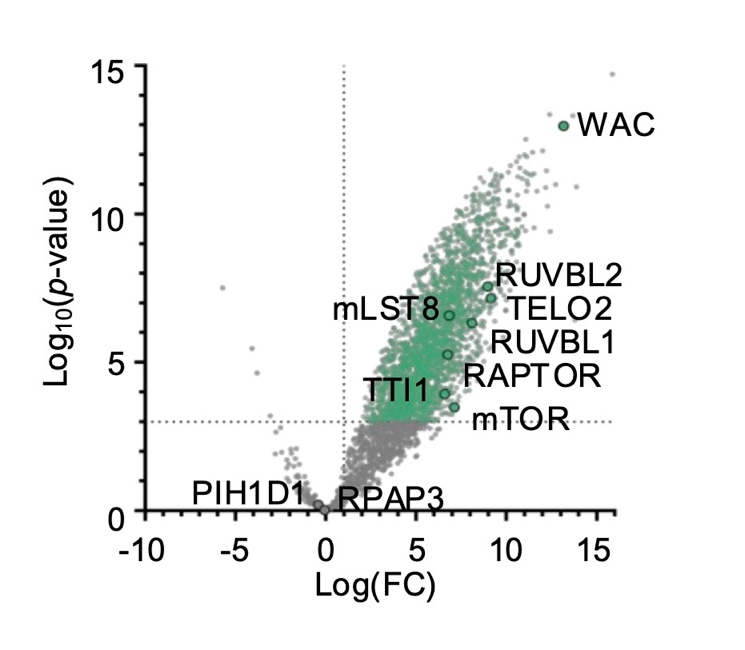
**

**Supplementary Figure S1. Mass spectrometry of WAC interactors in HEK293T cells using WAC antibodies.** (Volcano plot illustrating the distribution of all proteins identified by MS/MS after a WAC-Flag immunoprecipitation from HEK293 cells. Significantly enriched proteins (log_10_(p-value)>3 and log(FC)>1) are highlighted in green. Y-axis: -Log10(P-value)


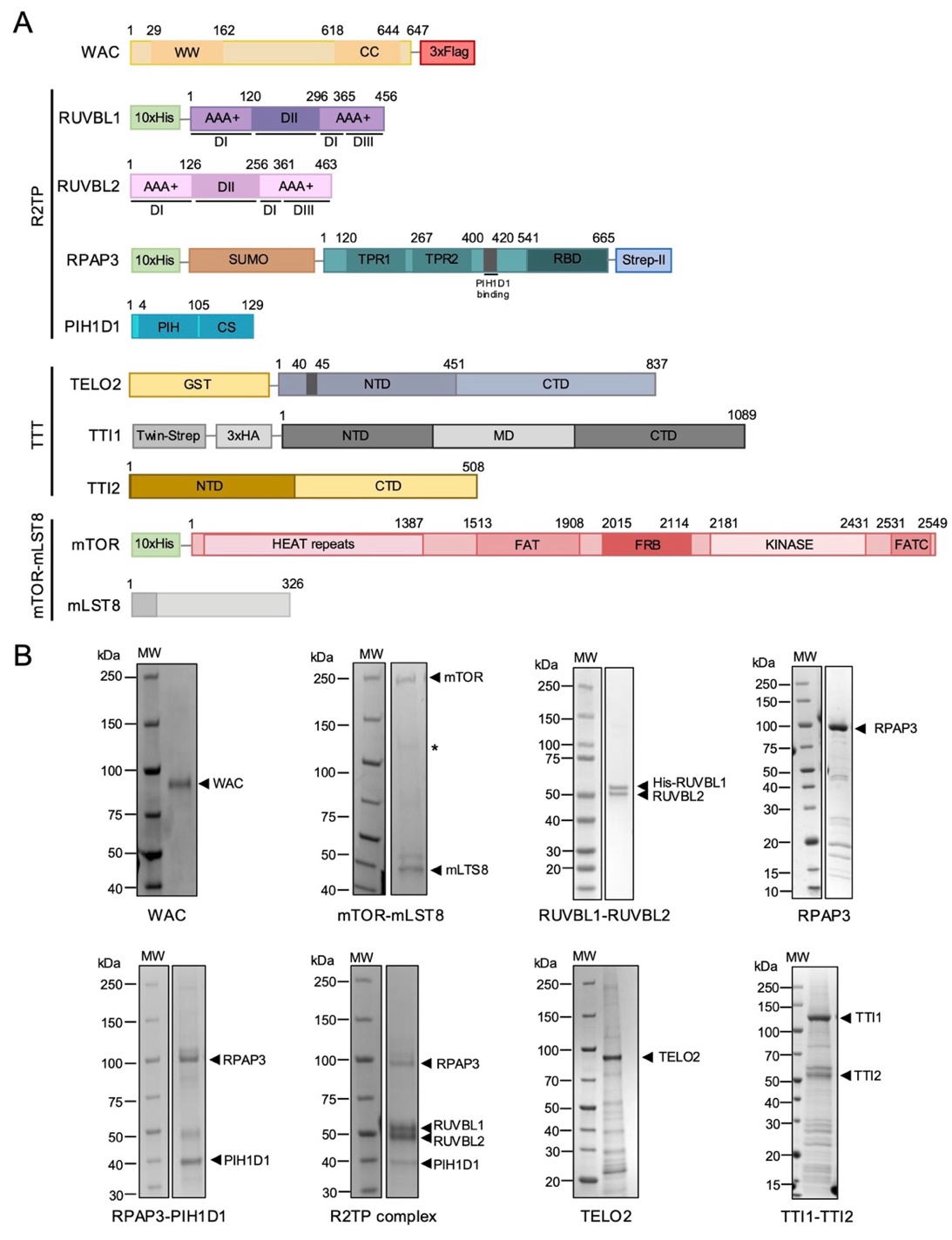


**Supplementary Figure S2. Purified proteins used in pull-down experiments.**

(A) Schematic representation showing the domain architecture of the proteins used in this work, WAC, RUVBL1, RUVBL2, RPAP3, PIH1D1, mTOR, mLST8, TELO2, TTI1 and TTI2. Residues for domains are indicated and specific Tags used for pull-down assays are shown in this panel, 3xFlag (red), 10xHis (green) and StrepII (blue).

(B) ﻿4–15% SDS-PAGE of purified WAC and the complexes used in this work, mTOR-mLST8, RUVBL1-RUVBL2, TELO2, TTI1-TTI2, RPAP3, RPAP3-PIH1D1 and R2TP, stained with QuickCoomassie (Generon).


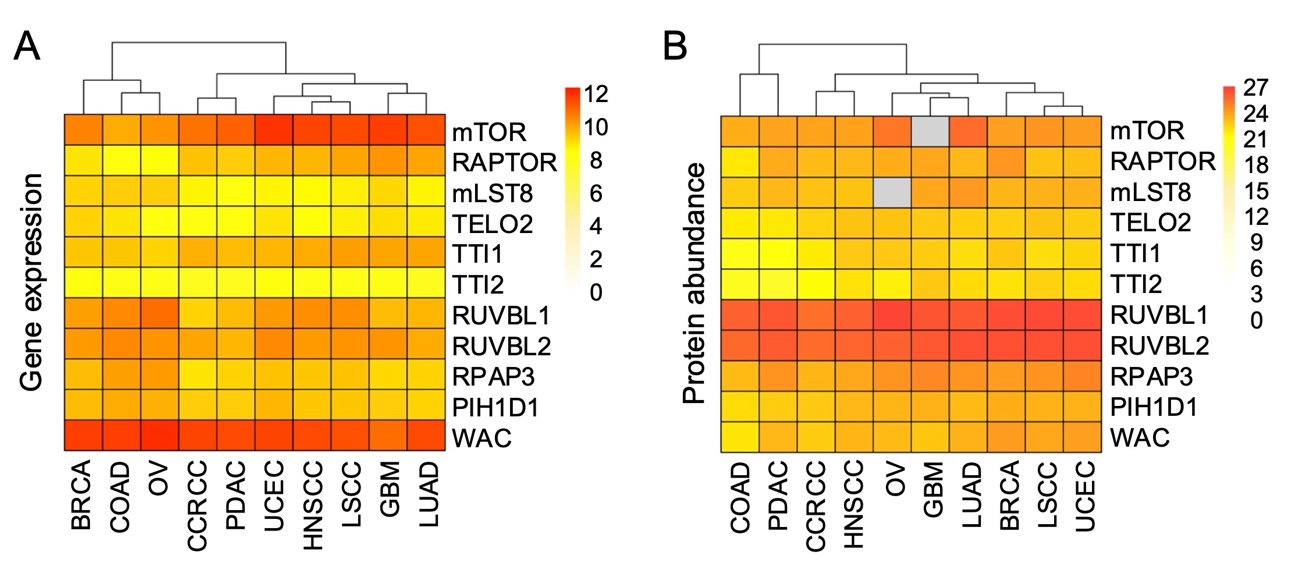


**Supplementary Figure S3. Gene and protein expression analysis of WAC-RUVBL1/2-TTT complex components across cancer types.**

Gene (A) and protein (B) expression profiles of the subunits of the mTOR-WAC-RUVBL1/2-TTT complex in 10 cancer types with CPTAC tumor samples. BRCA: Breast invasive carcinoma. CCRCC: Clear cell renal cell carcinoma. COAD: Colon adenocarcinoma. GBM: Glioblastoma. HNSCC: Head and neck squamous cell carcinoma. LSCC: Lung squamous cell carcinoma. LUAD: Lung adenocarcinoma. OV: Ovarian serous cystadenocarcinoma. PDAC: Pancreatic ductal adenocarcinoma. UCEC: Uterine corpus endometrial carcinoma.

**
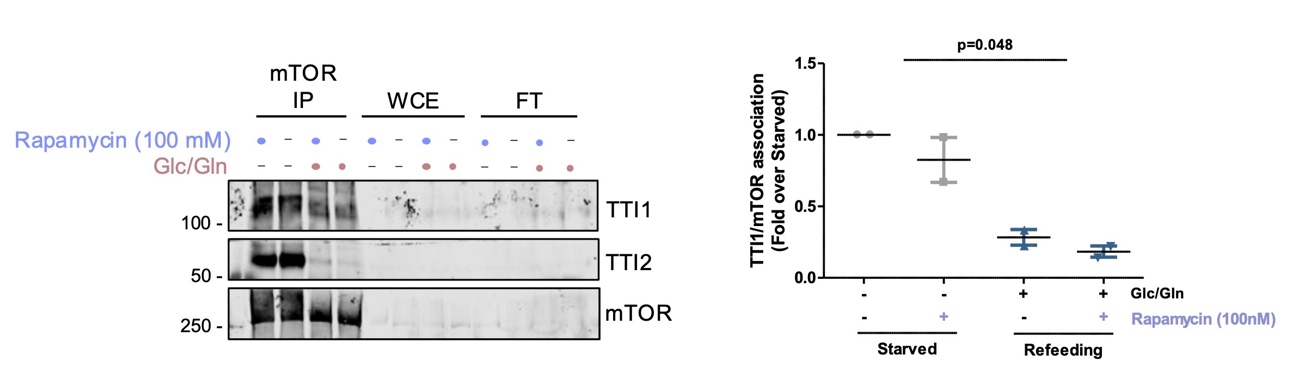
**

**Supplementary Figure S4. mTOR forms a complex with TTI1/TTI2 upon glucose and glutamine depletion.**

Immunoprecipitation of endogenous mTOR performed after to Glc/Glc starvation and recovery, in two conditions, either treating cells with rapamycin (30 min) prior to starvation and recovery, or without treatment with rapamycin. Data (mean ± SEM) were normalized by total mTOR immunoprecipitated and expressed as fold change of association with respect to the starved condition. Statistical significance was analyzed using two-sided unpaired t-test. p values, *p < 0.05. Representative blot of 2 independent experiments is shown.


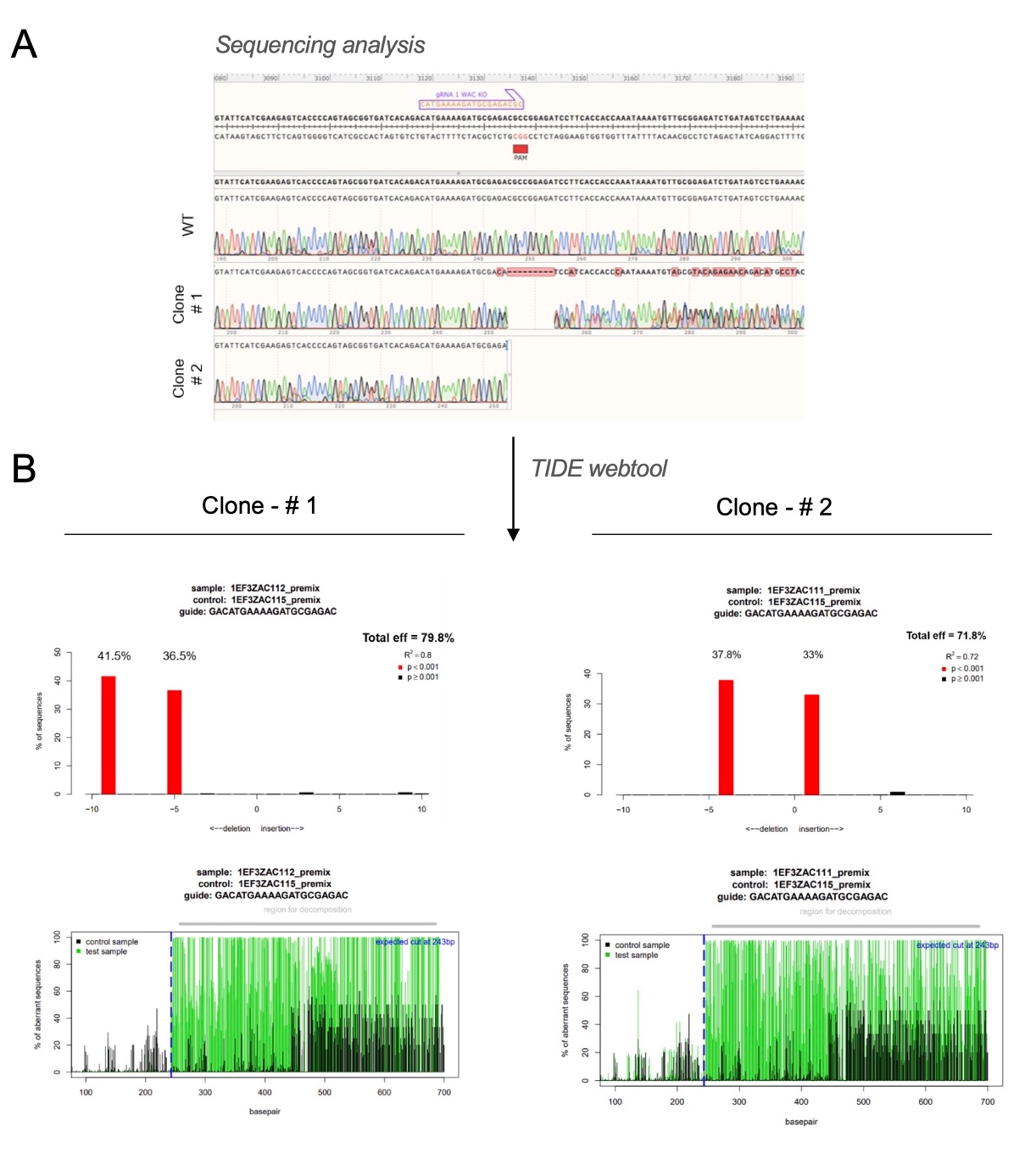


**Supplementary Figure S5. Characterization of WAC KO clones used in Figure 2.**

(A) Genomic DNA sequencing of WT and selected WAC KO clones was analyzed by using SnapGene software.

(B) Assessment of genome editing of two different WAC KO clones by sequence trace decomposition (TIDE analysis). Overview of TIDE algorithm and output, which consists of three main steps: (1) Visualization of aberrant sequence signal in control (black) and treated sample (green), the expected break site (vertical dotted line) and the region used for decomposition (gray bar); (2) Decomposition yielding the spectrum of indels and their frequencies; (3) Inference of the base composition of +1 insertions.

**Supplementary Tables**

**Supplementary Table S1. Oligonucleotides used for cloning.**

| **Construct** | **Name** | **Sequence 5’ - 3’** |
| --- | --- | --- |
| **pACEMam-WAC-3C-3xFlag**  **pACEBac-TEV-WAC-3C-3xFlag** | \| 3C_FW \| gccttcatccgtttccacggtgtg \| \| --- \| --- \| | CTGGAGGTGCTGTTCCAGGGAC |
|  | TEV_RV | GCCCTGAAAATAAAGATTCTCTCCGCC |
|  | WAC_FW | GAGAATCTTTATTTTCAGGGCATGGTAATGTATGCGAGGAAA |
|  | WAC_RV | CCTGGAACAGCACCTCCAGCACCATGAAGGAATTCTGATTTTTTA |
| **pACEBac1-GST-TEV-TELO2** | pACEBac1_GST_FW | GGCCTACGTCGACGAGATGTCCCCTATACTAGGTTATTGGAAAATTAAGGG |
|  | GST_RV | GCCCTGAAAATAAAGATTCTCTCCGCC |
|  | TELO2_RV | ATACAGGTCCTCCCGATCCGCAGACTAGGGAGACGCGGGTGGGA |
|  | TEV_TELO2_FW | GAGAGAATCTTTATTTTCAGGGCATGGAGCCAGCACCCTCAGAGG |
|  | pACEBac1_FW | GATCGGGAGGACCTGTATTAGCTCACTTGTCGCGGCCGCTTTC |
|  | pACEBac1_RV | CTCGTCGACGTAGGCCTTTGAATTCC |
| **pACEBac1-TwinStrep-3xHA-3C-TTI1** | G_block | GAGAATCTTTATTTTCAGGGCATGTGGAGCCACCCGCAGTTCGAAAAGGGAGGAGGATCTGGAGGAGGATCAGGTGGTTCAAGCGCGTGGTCGCATCCACAGTTTGAAAAGTACCCATACGATGTTCCAGATTACGCTTATCCGTATGACGTCCCTGACTATGCATACCCCTACGATGTACCCGATTACGCCCTGGAGGTGCTGTTCCAGGGACCT |
|  | G_Block_FW | GCCCTGAAAATAAAGATTCTCTCCGCC |
|  | G_Block_RV | TGGAACAGCACCTCCAGGGCGTAATCGGGTACATCGTAGGG |
|  | TTI1_FW | CTGGAGGTGCTGTTCCAGGGACCTATGGCAGTTTTTGATACTCCTGAGGAGG |
|  | TTI_RV | GCCGCGACAAGTGAGCTATCACTGCAGCTCCTTGAGCAGCTG |
|  | pACEBac1_FW | TAGCTCACTTGTCGCGGCCGCTTTC |
|  | pACEBac1_RV | GCCCTGAAAATAAAGATTCTCTCCG |
| **pACEBac1-TTI2** | TTI2_ FW | AAAGGCCTACGTCGACGATGGAGCTTGACAGCGCTCTGGAAG |
|  | TTI2_ RV | GCCGCGACAAGTGAGCTATTAAGTTCCATTGTAGGGTGCGCCTTCA |
|  | pACEBac1_FW | TAGCTCACTTGTCGCGGCCGCTTTC |
|  | pACEBac1_RV | CGTCGACGTAGGCCTTTGAATTCCG |
| **pACEBac1-TwinStrep-3xHA-3C-TTI1-TTI2** | Gene1_TTI1_FW | GTATGCAGCTGTCGACACCTGTATCGCCTAAGGTAGCGAGTTTAAACACTAGTATCG |
|  | Gene1_TTI1_RV | GTTGCAACTGAGCTCGTTACGTACAAGCAGCAGCCAACTCAGCTTCCTTTCG |
|  | Gene2_TTI2_FW | CTTGTACGTAACGAGCTCAGTTGCAACCCTAAGGTAGCGAGTTTAAACACTAGTATCG |
|  | Gene2_TTI2_RV | GGTCGCTGCTTAAGGTCAGATCAAGGCAGCAGCCAACTCAGCTTCCTTTCG |
|  | pACEBac1_Scaffold_FW | CCTTGATCTGACCTTAAGCAGCGACCCCACCGCTGAGCAATAACTATCATAACC |
|  | pACEBac1_Scaffold_RV | CGATACAGGTGTCGACAGCTGCATACGATACTAGTGTTTAAACTCGCTACCTTAGGAC |

**Supplementary Table S2. Amino acids and glucose concentrations in RPMI 1640.**

| **Component** | **Concentration (mg/liter)** |
| --- | --- |
| Glycine | 10 |
| L-Arginine | 200 |
| L-Asparagine | 50 |
| L-Aspartic Acid | 20 |
| L-Cystine | 65 |
| L-Glutamic acid | 20 |
| L-Glutamine | 300 |
| L-Histidine | 15 |
| L-Hydroxyproline | 20 |
| L-Isoleucine | 50 |
| L-Leucine | 50 |
| L-Lysine | 40 |
| L-Methionine | 15 |
| L-Phenylalanine | 15 |
| L-Proline | 20 |
| L-Serine | 30 |
| L-Threonine | 20 |
| L-Thryptophan | 5 |
| L-Tyrosine | 29 |
| L-Valine | 20 |
| D-Glucose | 2,000 |
